# Bending time: lessons from critical, community-engaged, liberatory research

**DOI:** 10.3389/frma.2023.1174694

**Published:** 2023-10-12

**Authors:** Brian D. Lozenski, Anita P. Chikkatur

**Affiliations:** ^1^Department of Educational Studies, Macalester College, Saint Paul, MN, United States; ^2^Department of Educational Studies, Carleton College, Northfield, MN, United States

**Keywords:** chronopolitics, participatory action research, community engaged research, racialized time, anti-colonial education

## Abstract

In this article, we use the framework of chronopolitics and racialized time to explore our experiences as professors of color at predominantly white institutions who strive to do emancipatory, community-driven research. Our shared work as organizers for Education for Liberation Minnesota (EdLibMN), a grassroots organization working to bring together various constituencies in Minnesota to organize for educational justice, led us to think together about chronopolitics as a framework to understand how our scholarly commitments to social transformation and liberatory education impact our labor and teaching practices at our institutions. This framework allows us to examine our relationships with communities in our individual research and advocacy contexts as well as in our shared work as organizers for EdLibMN. In particular, we explore how the urgency and timeline of our community-based advocacy work and the rhythms and improvisation of participatory action research are juxtaposed with the surveillance and evaluation of our labor and the urgency of “tenure clocks” at our institutions. We end by discussing our own transformational learning through our collaborations with community researchers and organizers. We speculate about the possibilities of bending time–the chronopolitics of collective struggle and joy–that allows us to focus on building relationships as a central tenet of emancipatory research practices and to ensure our own health and wellbeing as scholar-activists of color.

## Introduction

*It's a Saturday evening, and there are three of us, all Education for Liberation Minnesota (EdLibMN) organizers, playing UNO at one of our homes. Between slapping down cards, trash talking, and laughing, we're discussing some initial ideas for our “Let's Get Free” Saturdays programming, dreaming and scheming about how to create an intergenerational space for liberatory education*.

This mixing of pleasure, kinship building, and organizing work isn't unique to this night. In fact, it's this exact amalgamation of joy, community, and work that has made Education for Liberation Minnesota (EdLibMN) a space where both of us feel time bending as we breathe, laugh, and work in ways that feel a lot less possible in our professional lives as professors in predominantly white institutions.^1^ We use “bending time” as a way to describe the intentionality of transforming the affective, qualitative character of how we experience time. It has allowed us to put into practice the idea of centering relationships, something often mentioned as important in doing community-based, emancipatory research, but is hard to actually prioritize given the pace of the tenure-clock and other institutional timelines–the chronopolitics of white institutions. When we started thinking about how we incorporate emancipatory methods in our teaching and research, our shared experiences in EdLibMN became the focus of our conversations. Being a part of a grassroots organization led primarily by people of color and focused on liberatory education for all, and not just the privileged few that make it into our college classrooms, has been critical to our ability and capacity to be more of ourselves, to dream collectively, to recognize the urgency and relevance of our work beyond the deadlines and demands of academia, and to understand emancipation as a process and a practice, not a destination. It has allowed us to connect our teaching, research, and organizing in more powerful ways than if we were trying to do those activities alone. It has allowed us to reckon with Ruth Wilson Gilmore's poignant reminder about the impact of racism on our time: “Racism, specifically, is the state-sanctioned or extralegal production and exploitation of group-differentiated vulnerability to premature death” (Gilmore, 2007, p. 28). If we literally have less time as negatively racialized people^2^ in the U.S., being a part of EdLibMN has given us purpose and clarity about what we want to do with that time.

In this article, we use the framework of chronopolitics and racialized time (Cooper, 2017; Mills, 2020) to explore our experiences as professors of color at predominantly white institutions who strive to do emancipatory, community-driven research. As we allude to in the opening story, our shared work together as organizers for EdLibMN, a grassroots organization working to bring together various constituencies in Minnesota to organize for educational justice, led us to think together about chronopolitics as a framework to understand how our scholarly commitments to social transformation and liberatory education impact our labor and teaching practices at our institutions. This framework allows us to examine our relationships with communities in our individual research and advocacy contexts as well as in our shared work as organizers for EdLibMN. In particular, we explore how the urgency and timeline of our community-based advocacy work and the rhythms and improvisation of participatory action research are juxtaposed with the surveillance and evaluation of our labor and the urgency of “tenure clocks” at our institutions. We end by discussing our own transformational learning through our collaborations with community researchers and organizers. We speculate about the possibilities of bending time–the chronopolitics of collective struggle and joy–that allows us to focus on building relationships as a central tenet of emancipatory research practices and to ensure our own health and wellbeing as scholar-activists of color. We are hoping to speak to other academics in similar circumstances. Can we indeed change the quality of our time in community, in solidarity, in collectives, and not just as individual acts of refusal and resistance?

## Chronopolitics of the university and the community

Exactly, so we—we ain't got time to waste time, my nigga

Niggas gotta make time, bro

The judge make time, you know that, the judge make

time, right?

The judge make time so it ain't shit

-Kendrick Lamar “i” (Duckworth et al., 2014).

The exchange above takes place in the song “i” from Kendrick Lamar's critically acclaimed (2014) album, *To Pimp a Butterfly*. On the album version of the song, Lamar has his on stage performance interrupted by an altercation in the crowd. Lamar stops the music to address the audience directly and insinuates that there have been too many lives in his immediate circle lost to violence. He suggests that because so many of his close relations had their lives cut short that his community needs to “make time” by not engaging in horizontal violence. His plea then links to the broader judicial system by highlighting how jail sentences can be understood as “making time.” In this brief interlude, Lamar highlights the complexities and interlocking layers of what Mills (2020) describes as chronopolitics, where “the social life of time will be intimately entangled with the political life of time” (p. 298).

As seen in Lamar's assessment, chronopolitics extends past personal considerations of time, as measured by one's lifespan and day to day activities, to the political dimensions of time, where structures, such as the carceral state, dictate how time is spent, infusing a qualitative essence to time. In our analysis, we consider the implications of time from micro (personal) to macro (structural) dimensions, while emphasizing what we experience as a meso (intermediary) dimension of time that exists in our scholar-activism. This meso timescale is defined by the ways that institutional markers of time in university spaces (e.g., tenure clocks, semester schedules, credit hours, graduation) come into contact with community markers of time (e.g., urgent social issues, relationship building, issue-based campaigns). More often than not, the chronopolitics of the meso layer are contested, contradictory, and incommensurable, requiring scholar-activists, particularly those from racially marginalized backgrounds, to make calculated decisions about personal wellbeing, participation in broader social change, career advancement, and scholarly pursuits.

Before exploring the nuances of the meso level, we provide an overview of macro and micro chronopolitics drawing from the work of critical scholars such as Mills (2014, 2020); Cooper (2017), and Mahadeo (2019). Mills, a racial theorist and philosopher, provides a succinct overview of macro chronopolitics, reminding us that time can be understood as collective social memory (Zerubavel, 2003); as a capitalist measure of efficiency and labor (bourgeois time) (Marx, 1976); as a historical marker of coloniality through land domination and the dispossession of epistemic forms of timekeeping; or as a racialized outgrowth of coloniality that denigrates non-white understandings of time (e.g., colored people time) (Hanchard, 1999). According to Mills (2020), Chronopolitics has to do with the multiple different ways in which power relations between groups–whether formally acknowledged in recognized systems of governance or not–affect both the representations of the relations between these groups and the world, in their specifically temporal aspect, and the material relation of these groups to the world, in their specifically temporal dimension (p. 299).

These chronopolitics are obvious, yet hard to discern because they structure so much of our lives: “I'm late for work/class/appointments.” “There are 365 days in a year.” “I wish I had another hour in the day.” “Time is money!” “I can't wait to turn 18 and become an adult.” Mills reminds us that we currently live in (Euro) Christian time marked by the life of Jesus, hence our writing of this article in 2023 (AD). These historical “clocks” are at the heart of much of the historical and contemporary educational justice work attempting to reclaim the chronopolitics of history. When do we start the historical study of the Americas and what has become the United States? 1492? 1619? 1776? 10,000 B.C.?

Sociologist Mahadeo (2019) applies the work of Mills and others by exploring a set of suppositions about the nature of racialized time. He posits,

Rather than using an adjective (i.e., what), it may be more sociologically productive to use a determiner (e.g., whose). Asking “Whose time is it?” alludes to the inherent power relations associated with time that privilege some at the expense of others. In reframing the question, we open possibilities to appreciate the way time is experienced differently between individuals and groups (p. 186).

Mahadeo explores how youth of color, and black youth specifically, experience racialized time, concluding that processes of negative racialization literally compress time. This compression is not only about time as quantity, but also quality. He considers the perspectives of youth of color who believe that the quality of their time is degraded because of the incalculable amount of “time required to process experiences with racism, racialization, and discrimination, literally and figuratively, don't count” (p. 196). The cognitive “labor” expended as a function of negative racialization is not considered in the daily calculation of time spent, resulting in ghostly chronic haunting of communities of color.

Black feminist scholar Cooper (2017) continues this line of thought connecting the macro to the micro, the political to the personal, and the theoretical to the mundane. Cooper describes how racialized time literally constructs time as white property. Cooper suggests that black people “experience time discrimination… not just as structural, but as personal: in lost moments of joy, lost moments of connection, lost quality of time with loved ones and lost years of healthy quality of life.” The structuring of our personal lives in and around macro chronopolitics accounts for the affective experience of time. It is in this interplay between macro and micro chronopolitics that we situate our exploration of community-engaged liberatory praxis at the meso-level of institutions and communities.

## Navigating the contradictory chronopolitics of the university and the community

Contemporary universities are historically mediated by the macro chronopolitics of bourgeois time, settler colonial time, and racialized time. These institutions play a vital role in the processes of instilling the “collective social memory” (Zerubavel, 2003) of the settler nation-state through the construction of what counts as legitimate knowledge. They adhere young people to the logics of bourgeois time through the accumulation of credit and debt (Harney and Moten, 2013)—concepts that are inherently linked to time. Universities function through strict metrics of bourgeois time by literally assigning clocks to learning. In colleges around the country, students pay by the credit hour to be taught by faculty who are considered “full time” or “part time.” Within the hierarchy of the university faculty, like us, are determined by the institution to be on the tenure-track, tenured, fixed-term, or adjunct. The mathematics of non-tenure track faculty are literally calculated by the credit hours they are responsible for. Tenure-track faculty are placed on a “clock” through which they have a certain number of years to demonstrate they have earned tenure based on their research and scholarship. The tenure clock sets the “pace” for research and academic publication, which are activities that are harder to time-manage than teaching, especially when it involves community collaborations. The chronopolitics of the university are highly visible and easy to discern and measure.

The same cannot be said for community-engaged scholarship. However, one defines “community,” it is mediated by relationships, whose chronopolitics are hard to measure and determine ahead of time. Communities (and relationships) can be formed through myriad circumstances including but not limited to residential proximity, kinship, cultural familiarity, language, specific skills or practices, and/or political unity. The chronopolitics of various communities can be wildly different with regard to the nature of, and motivating factors for, relationships with academic institutions. Cushing-Leubner et al. (2021) describe the mythology of knowledge production for the academic archive as related to a production model of keeping pace with the tenure clock. They argue that this mode of knowledge construction is antithetical to more communal and relational forms of teaching and learning. For the purpose of this analysis, due to the nature of our scholarship, we will emphasize political unity and liberatory educational practice as the impetus of our community building in EdLibMN. The chronopolitics of political struggle are often about organizing against the very formation of the macro chronopolitics of bourgeois time, settler colonial time, and racialized time that inform university chronopolitics.

In our research, we seek to understand and illustrate the historical and contemporary roots of educational dispossession that result in unequal and often violent schooling experiences for Indigenous youth and youth of color (Chikkatur, 2021; Lozenski, 2022). Ironically, the communities we seek to build political solidarity with through our teaching, scholarship, and activism are often those who have been locked out of the very institutions we inhabit as faculty. Although our labor is situated in university space, our research often critiques the logic of these spaces and how they inform and structure colonial preK-16 schooling environments in the name of meritocracy, access to wealth, and social hierarchy (Lozenski, 2017; Chikkatur, 2019), all while legitimizing epistemic ignorance (Calderon, 2011), carceral enclosure (Sojoyner, 2016), and racialism (Robinson, 2020). Our critiques come out of our sense of obligation to work collectively to better the material conditions of the communities that nurtured us as children and scholars. As scholars of educational contexts, we would believe that it would be unethical of us *not* to work to change those contexts that harm young people.

The community-engaged nature of our research compels us to not only consider the competing chronopolitics of the university and our communities, we must also consider the chronopolitics of research methodologies. Universities seek to be arbiters of research through the technologies of peer review. Peer review functions as a disciplinary force both in processes of tenure review and the dissemination of research through academic publication (which is a key feature of tenure review). What gets constructed as “scholarship” for academics is largely determined by other academics and bounded by disciplinary histories. The chronopolitics of research are structured through recognizable methodologies that typically follow the pathway of the creation of questions, research design, execution, analysis, and dissemination through peer-reviewed publications. Methodologies, whether qualitative, quantitative, or mixed, regiment researchers' time within this process. However, when faculty seek to interrupt these chronopolitics by redefining who counts as a peer or even a researcher, the legitimacy of their research methods and analysis can come into question by the university.

In our attempts to shift the chronopolitics of methodology, we first recognize that the framing of scholarship is, in and of itself, a labor/time construct for faculty. At its most basic level, “scholarship” is a designation of time spent. The most recognizable time is supposed to be spent reading, collecting data, analyzing, and writing. The chronopolitics of community-engaged methodologies recognize that time should also be spent building relationships, sharing stories, and determining that a different set of “peers” can be the arbiters of the research or researchers themselves, such as a group of mothers, community elders, or a task force created to implement a co-constructed policy. These are examples of bending time toward an emancipatory chronopolitics that differently structure research methodologies and expand notions of legitimate scholarship, which is really about how we spend our time.

In the analyses of our particular research and advocacy contexts that follow, we highlight our attempts to exist in the meso chronopolitics of community-engaged liberatory research and organizing. This meso layer is an inherently contradictory space where our labor as faculty comes into conflict with our community-building and praxis as organizers. We describe our attempts to resist, refuse, and even bend white bourgeois time in the service of movement building and working against the chronopolitics of our respective institutions. Readers may ask why we would choose to “play our hand” in this way, revealing our resistance to university chronopolitics. While there is a risk in divulging our insurgent chronopolitical stance, we believe there is the possibility for generative outcomes in bringing the discourse of chronopolitics in scholar-activism to light. We also recognize that universities benefit from, and measure our worth, largely through our traditional scholarship, including these forms of counterhegemonic publications. In previous writing, Lozenski (2021) has developed the metaphor of critical faculty of color as trains used to “carry precious knowledge from communities to the academy” (p. 78). Universities do not care what type of knowledge they accumulate, only that they are the holders of the commodity of legitimate knowledge. In Anita's case, while some of these critiques did lead to uncomfortable interpersonal dynamics between her and her colleagues, she was still able to use her experiences to gain recognition using the currency of academia by publishing about them (Chikkatur, 2019)! In the twisted logic of the settler colonial higher education institution, our insurgent/critical/counterhegemonic scholarship is useful to these institutions because they get to bolster their image as critical institutions while also reifying the status quo. In what follows, we articulate our own experiences as community-engaged scholars attempting to steal/borrow/transfer labor across a chronopolitical spectrum from the university to the community.

## Personal journeys and experiences

How many times do I sit and listen to ya?

I don't know why I keep sittin' listenin' to ya

It's not that I'm bored

I just heard it before

The space you take up

The curve of your learning

That's my labor, my love

Explaining myself again

I could have run a mile instead

I could have twist my ends instead

I'm laid out in bed

Gaslight in my head

But I said what I said

- Jamila Woods “Eartha” (Woods, 2019).

### Brian

In November 2022, I took the podium at a faculty meeting during an open discussion of a motion to include more supportive language of community-engaged scholarship (CES) in the faculty handbook. I had been working with a group of faculty and staff for a period of 3 years to document ongoing CES efforts, research how other colleges support this form of scholarship, and host open forums to create dialogue on campus. As I stared at my colleagues over the podium, I admit that I was exhausted and frustrated that I, once again, needed to plead this case. I began by describing that a large part of my scholarly agenda had to do with documenting and analyzing Ethnic Studies-based pedagogies in K-12 schools and community-based organizations. I then turned to the right-wing attacks on Ethnic Studies that have gained more traction under the guise of recent anti-Critical Race Theory (CRT) legislation, but have always haunted the liberatory ethos of the modern Ethnic Studies movement beginning in the late 1960s. I asked my colleagues if the unit of analysis of their research had ever been under attack.

I described how the very existence of my work was under threat and how this reality compelled me to engage in forms of scholarly-activism with broader communities of color in Minnesota. I described the amount of time I spend organizing with EdLibMN and the Minnesota Ethnic Studies Coalition to provide grassroots support for Ethnic Studies teachers and also to author and lobby for policies that would protect the rights of these educators. I explained that my expertise was necessary to help develop the licensure standards and pathways for the first ever Ethnic Studies teaching license in Minnesota history. I also explained that authoring language for a teaching license would not count as scholarship under the handbook language to which I was subject.

While we did, overwhelmingly, pass the motion to change the handbook language at the subsequent faculty meeting, I do not believe I was able to effectively convey the chronopolitics of my scholar-activism. The dogged battle for Ethnic Studies in Minnesota schools has been happening for nearly half a century (Lozenski and Hamilton, 2020), which reflects the broader national struggle for Ethnic Studies at the K-12 and post-secondary level since the general strike at San Francisco State University in 1968 (Ferguson, 2017). This protracted struggle notably came to a head with the banning of the Mexican American Studies program in Tucson in 2010 (Cammarota et al., 2014), which set the tone for the most recent legislation in places like Florida. Following the lead of youth activists (Lonetree, 2021; Youth4EthnicStudies, 2023), the most recent iteration of the fight has managed to gain some traction with the formation of the Minnesota Ethnic Studies Coalition in 2019, composed of nearly 20 educational justice organizations. While EdLibMN is not a policy or lobbying organization, we joined the Coalition and have taken leadership roles in several of the sub-committees. The building of an effective coalition is not an overnight process. We have spent countless hours developing the coalitional infrastructure, while directly agitating the state legislature, governor's office, department of education, and educator licensing board to support proactive Ethnic Studies policies. The effort has been grueling, and has often come in conflict with my institutional expectations for scholarly output in the form of peer-reviewed journal publications. In the work of the Coalition, “peer-review” takes the shape of the bureaucratic structures of the State, such as legislative rulemaking, to which the policies and licenses we offer are subject. My co-researchers are Ethnic Studies teachers, youth, and community practitioners who have deep expertise in Ethnic Studies pedagogy and practice. This shift in my peers necessitates a shift in my practice as a scholar away from the disciplining practices of the College where I teach.

The chronopolitics of the Coalition have varied with context. The off-beat rhythms of the political process have pushed us to have urgent late-night meetings to respond to public and personal attacks against our work, to use group texting platforms to strategize through hundreds of texts, to mobilize our base to attend and offer testimonies at “public” forums, to literally disrupt business meetings in which our needs were being ignored, and to be available for last minute invitations from reporters, podcasters, and radio personalities. In 1 week, we may have to lobby a moderate Democrat on the House Education Committee to support a bill for an Ethnic Studies graduation requirement and then come together to strategize how to support a colleague whose address has been posted on a right wing social media platform.

There is an eerily appropriate parable to represent these exhausting chronopolitics, in which an enslaved person is told to bring a note from the plantation where he is enslaved to the next plantation and show it to the owner. The next slave master scribbles a new note and sends the enslaved man to another plantation with urgency. After several rounds of this same experience, the man finally runs into another enslaved man who could read. When he asks to know what the note says, he is told it says “keep the nigger running.” The parable shows that time as white property can also be weaponized into a game of time-wasting for communities of color. While I am not suggesting that the work of the Coalition is in any way a waste of time, I am arguing that we are subject to white time as property, and that our work to change the laws of Minnesota have put us in a position of reaction to dominant forms of chronopolitics. Mahadeo's assertion of “whose time” resonates with the experiences of the BIPOC-led Coalition who have had to spend time that “doesn't count” in our lives as educators, parents, and activists. We volunteer our time to improve the lives of youth in our communities, and to live in the tradition of Ethnic Studies. These exchanges of time are often confusing, incalculable, and disorienting to our universities and to many of our peers.

### Anita

Between 2018 and 2022, I was involved in a federal grant funded participatory action research (PAR) project in Faribault, MN, focused on investigating the lower rates of high school graduation rates for Somali and Latinx students compared to their white peers. I had the opportunity to work with numerous community research teams of Somali and Latinx youth, Somali and Latinx parents, and white administrators and educators. I also had the opportunity to work closely with two Latinx students at Carleton, who have been my main research collaborators for developing a website about the project and about PAR in general (Carleton-Faribault PAR Collaboration, 2023). As I noted in a recent book chapter reflecting on how my various identities and experiences have mattered in this project,

As a member of this project, I have tried to stay mindful of how the opportunities and successes I have had in my career have been deeply shaped by my race and class privileges, while staying attuned to the similarities that I share with the community researchers as a way to build reciprocal relationships across differences in age, race, gender, class, language, professional status, and migration experiences. Like the Latinx and Somali parents and students, I am an immigrant and a child of immigrants. Like the education experienced by most of the White educators at the high school, my education has been indelibly American, White, and middle class in its orientation. Like the Latinx and Somali parents and students, experiencing and witnessing racism have been life-shaping and life-shattering experiences for me as a person of color. Like the White educators, English is my best language, the one that I think and dream in. Like some of the Latinx and Somali youth, I am somewhat fluent in my native tongue (Kannada), understanding more than I can express. Like some of the White educators, I am attempting to learn Spanish and Somali as one way to be better at my job (Chikkatur, 2022, p. 42).

Much more so than as a professor at a predominantly white institution, this project has allowed me to be more of myself and to draw on more of my life experiences as I work across multiple languages, contexts, and communities. Whatever I've brought to the project, though, I've received back so much more. This work, and my work organizing with EdLibMN, have provided me with spaces to do the work of racial equity in education with people and communities of color. Having regular meetings with the EdLibMN core organizing committee and planning and attending the BIPOC Ethnic Studies Teacher Mentoring Network gatherings provided me a space of refuge and connection during the pandemic, especially during and in the months following the racial justice uprisings in the Twin Cities. Working with Latinx and Somali parents and youth in Faribault has been similarly rejuvenating. This work has allowed me to focus on my own wellbeing and sanity while working at a predominantly white institution. In her TED talk, Cooper (2017) notes,

Those in power dictate the pace of the workday. They dictate how much money our time is actually worth. And Professor George Lipsitz argues that white people even dictate the pace of social inclusion. They dictate how long it will actually take for minority groups to receive the rights that they have been fighting for.

I have become less and less engaged and interested in doing the work of “inclusion” within a predominantly white institution–I do not want to let others “dictate” my sense of belonging and inclusion. While I can't completely overturn or escape the chronopolitics of the institution, I can perhaps use the privilege of tenure to reconfigure at least some of my time. And time is essential when engaging in PAR research; it takes time to build relationships of trust and a sense of community, a central tenet of the PAR process. When my co-PI, Emily Oliver, and I first started facilitating community research team meetings, we noticed the “check-in” part of our meeting agendas, especially with Latinx parent team, often ended up taking 45 min to an hour of our weekly 2 h meetings. Initially, we worried about not having time to get through all the items on our agenda; however, we realized that the time that the parents took to share what was happening in their lives and their children's lives were part of the data for their research question around parents' roles in schools–through these stories, we were able to see patterns in what supported or what got in the way of their ability and capacity to connect with their children's schooling. While my co-PI and I both believed in the ethics and approach that PAR work necessitates, it wasn't until we let go of our deeply ingrained ideas of what meeting time should entail that we learned to lean into the legitimacy of the chronopolitics of participatory research. Because the participatory process involves deep collaboration, research teams need time to develop community norms and democratic decision-making processes. Since the problems being investigated in these projects are often deeply personal for those involved, creating trust ensures people feel safe enough to be honest and open about their experiences and ideas. These experiences and ideas are crucial building blocks of developing more emancipatory methods of investigating problems within communities by communities.

As I continue my collaboration in Faribault, I am committed to use my time and the resources of my institution to work with comrades outside of the institution to create emancipatory spaces, and perhaps even just emancipatory moments, for young people and adults of color. This move is also one of self-preservation: spending my time finding those spaces where I'm already valued and included means I'm not wasting my time begging to be valued and included in an institution that was never built for me. Rather than struggling for inclusion on an individual basis, I want to engage in a collective struggle to push back against frameworks and structures that make emancipation impossible. My community-based work has given me strategies and opportunities to push back against the lens of deficit through which universities tend to see communities of color; against the idea of universities being the benevolent helpers of “downtrodden” communities and having the burden to leading into civilization those with the capacity to progress” (Mills, 2020, p. 307); and against even how institutions see their own students who are from “downtrodden” communities. The narrative about “including” students from communities who have been historically denied access to institutions of higher education is all about social uplift and not social upheaval! As García Peña (2022) argues, the “project of ‘diversity and inclusion”' at universities often means bragging about the “‘kids from the ghetto'…because it facilitates the narrative of the white savior” (p. 49). The “social mobility” purpose of universities is about helping these students gain the knowledge and the networks to become part of the elite, rather than dismantling the system that produces an elite.

Reckoning with that fundamental truth about our institutions has meant that I've chosen to spend less of my time in trying to reform my institution from within; though as Brian's efforts demonstrate, asking our institutions to recognize and honor our community-based work is important. Given “the minority tax and erasure of our labor” as scholars of color (García Peña, 2022, p. 39), we do need to “insist that our labor becomes visible, acknowledged, and compensated” (p. 51). At the same time, we “need to find our people and together we need to take the resources available to us through our institutions and use them to build spaces that sustain us” (p. 51) and for me, that latter task has been the focus on my post-tenure professional life.

Being involved in the PAR project and EdLibMN has allowed me to ask different questions about my teaching and research and how my classroom and research projects can contribute to the collective, community-driven project of emancipation. As a faculty of color at an institution with mostly white faculty and students, I am grateful to have a network of educators, youth, and parents of color who help me envision more liberatory ways of teaching, learning, and researching. My conversations with this network have taught me the importance of building life-affirming classrooms, following Ruth Wilson Gilmore's and other abolitionists' insistence that abolition is not only about the dismantling of oppressive systems, including prisons and police, but also about the everyday work of building transformational ways of relating to each other (Kaba, 2021; Purnell, 2021; Gilmore, 2022). I have come to learn that while I may not be able to transform the macro-structure of chronopolitics, perhaps collectively we can change the quality of time in our day-to-day lives, one UNO game at a time!

##  Bending time: what we have learned from our work in/with communities and each other

Individually, we have strategized differently about how we negotiate our time and presence within and across our institutions and about how we can bring in resources from multiple institutions to support our community work. These strategies are related to our specific racial, ethnic, gendered, and other social identities as well as our particular histories and experiences with schooling inside and outside the U.S. However, what brings us together is our shared work as organizers with EdLibMN. Sharing our individual strategies with each other has led us to appreciate what we have done and can still do even within the constraints of institutional chronopolitics. We want to remind ourselves (and our readers) that we do have a level of privilege because of tenure that allows us to approach our teaching and research in more emancipatory ways and that we have the capacity to repurpose some of the expectations for how our institutions want us to spend our time. As we noted earlier, this very article is an example of literally showing our hand: the university cannot care less about our critiques couched as it is in academic prose, the publication looks good for us, it might help us get a promotion, we continue to get praised for our work…While we cannot entirely escape or upend the chronopolitics of our institutions as individuals or even as members of a grassroots organization, we can perhaps find ways to change our experiences of those chronopolitics through our engagement with each other and in our communities. While Mahadeo compels us to change our question from “what time is it” to “whose time is it,” the chronopolitical environment of EdLibMN allows us to ask, “*How* is the time.”

### How is our time?

One of our dear EdLibMN comrades, MK Nguyen, a powerful organizer and leader in the Southeast Asian community in St. Paul, constantly reminds us to be attentive to the “how” of our time. MK is attuned to the process work of EdLibMN, ensuring that all voices are uplifted, that children remain centered in our work, and that we do not get bogged down in the drudgery of liberal politics. She asks us questions like, “how is your heart,” “what are we visioning,” and “who are we serving.” While these questions are situated in the present, they offer a way to practice a liberatory politics that transcends the current moment, a chronopolitics that can exist in a world in the making. MK's questions introduce a different set of metrics for how EdLibMN spends time. While we are interested in contending with the educational structures that are detrimental to our families and communities, the work that actually serves our souls lies in creativity and building educational environments that do not yet exist. In building these environments, we try to remain attuned to healing from intergenerational trauma and also critical joy. Critical joy is about the feeling of excitement and elation from the endorphin rush that comes from collective creation and struggle toward liberatory futures. These forms of joy are critical because they are not simply about having fun. They are embedded in projects of resistance to dominant supremacist models of education, and imagining otherwise. As Kelley (2002) argues in *Freedom Dreams*, “Revolutionary dreams erupt out of political engagement, collective social movements are incubators of new knowledge” (p. 8). This space of knowledge incubation is inherently joyful.

One of EdLibMN's central projects has been participating in the MN Ethnic Studies Coalition, which Brian mentioned above. EdLibMN has taken a leadership role in several of the sub-committees of the coalition. One of the things we noticed during the protracted effort to insert Ethnic Studies as a central aspect of Minnesota's Social Studies state standards, was that some of the people engaged in the early struggle were not able to maintain their commitment to continue to show up each week. Unlike some other coalition members, EdLibMN folks had a space of critical joy to heal, reflect, and rejuvenate themselves to go back to battle. *How* we spend time in EdLibMN impacts *how long* we are able and willing to sustain ourselves in the negative and exhausting space of antagonistic political work. The political enactment of critical joy is ultimately about sustainability in political struggle through affirmation, healing, elation, and preparation.

Thinking about the *how* of our time also helps us place ourselves in the emancipatory traditions of resistance and refusal coming out of Black Studies/Ethnic Studies/Indigenous Studies (Grande, 2018; Kelley, 2018; García Peña, 2022). EdLibMN historicizes our work, recognizing that the battles we fight did not begin in our lifetime and will not end when we pass. Community engagement is a central tenet of these fields, given the histories of how they became part of universities in the first place through student demands and the mobilization of community knowledges and power (Ferguson, 2017; NPR, 2020; García Peña, 2022). As García Peña (2022) notes, “Social engagement and community-grounded learning have always been at the center of ethnic…studies efforts in the United States” (p. 72). In his love letter to black activist students, Kelley (2018) writes, “There is a long history of black activists repurposing university resources to instruct themselves and one another–to self-radicalize, in effect” (p. 154). As scholars building specifically on traditions of Black Studies and Participatory Action Research, we would betray these traditions if we were not doing this work of community organizing and trying to shift the chronopolitics of the university to meet our communities' needs such as the broad adoption of Ethnic Studies in K-12 schools or the development of culturally responsive school cultures that affirm all students. We also recognize the long history of resistance within negatively racialized communities, outside of academia, toward agentic chronopolitics, such as work slowdowns by enslaved black women (Davis, 1971; White, 1983). As Quecha scholar Grande (2018) powerfully reminds us: We “need to *commit to collectivity*…The journey is not about self–which means it is not about promotion and tenure–it is about the disruption and dismantling of those structures and processes that create hierarchies of individual worth and labor” (her emphasis, p. 61). The collectivities we are committed to are not just within academia but also in our communities. We want to push back/bend the chronopolitics of the university so as to make room for a different chronopolitics that allow for more emancipatory research methodologies. We want to be cautious, however, about how much pushback can happen on an individual level. As Schleck (2022) importantly reminds us, time and resources even for more traditional research as well as the academic freedom necessary to pursue more emancipatory research methods is being steadily eroded through the adjunctification and atomization of the professoriate.

Considering the reality of white time that leads “premature death” (Gilmore, 2007) and to lost moments of joy and connection, lost time with loved ones, and lost years of health and quality of life (Cooper, 2017) for black and other negatively racialized people, our work with EdLibMN that allows us to resist and bend time through joy, connection, and quality time with comrades committed to the same struggle toward liberation becomes important in the sense that we may not be able to prolong our lives, but we can prolong our ability to stay in the struggle and not get burned out. Our time spent in EdLibMN spaces helps us heal and it helps us to both shift the quality of our time as scholar-activists *and* to lengthen the time we're able to be *activists*. To those of you reading this article, especially scholars of color, who are navigating similar tensions trying to work within academia and within communities, we hope that you, too, have been able to find such collective spaces of renewal and rejuvenation. We believe these spaces to be particularly crucial for junior faculty, graduate students, and contingent faculty to find courage and comradeship in community. Gilmore's notion of “premature death” is speaking to a population-level measure of the impacts of racism–not some kind of prediction about any of our individual lives. And we do this work of collective struggle to change that population-level parameter of wellbeing and health, to do our part in trying to bend that long arc of the moral universe toward justice (King, 1968) and to do it in a way that transforms the quality of our time *and* what we do with that time.

## Data availability statement

The original contributions presented in the study are included in the article/supplementary material, further inquiries can be directed to the corresponding author.

## Author contributions

All authors listed have made a substantial, direct, and intellectual contribution to the work and approved it for publication.

## Footnotes

^1^To learn more about Education for Liberation Minnesota, how we got started, and our current work, please visit our website: https://www.edliberation.org/chapters/edlibmn/.

^2^We use the phrase “negatively racialized people” to mark that white people are also racialized, but in ways that benefit them and their material realities. Furthermore, throughout this article, we use and cite language (e.g. “the n word”) in various ways. We understand that all language is political and subject to the interpretations of readers who have myriad sociopolitical locations. Our use of language that may be deemed “controversial” is for the purpose of accuracy and for creating the maximum context possible within the space we have.
